# Specific Treatment of Diphtheria and Croup

**Published:** 1884-09

**Authors:** 


### Specific Treatment of Diphtheria and Croup.

Dr. George A. Seynn, of Monongahela City, Pa., read a paper at the last meeting of the American Medical Association (*Jour. Am. Med. Association*) upon this subject. He recommended bichloride of mercury as a specific for diphtheria and chloride of gold for croup.

The bichloride of mercury should be used in the first stage of diphtheria, and in large and frequently repeated doses, and not after everything else has failed. The effect of large doses of this remedy in the early stage of the disease is to reduce the temperature, relieve pain in the head, back and limbs, unlock the secretions, lessen the soreness in the throat in time to relieve nausea and vomiting, restore appetite, and, most of all, to prevent the generation of the poison in the membrane, and to check the formation of the membrane, or to cause it, if formed, to speedily disappear. It is best given in solution, so that when excessive nausea is present, the dose may be gradually lessened and the time shortened, giving the stomach a chance to dispose of it, but at the same time keeping up full treatment.

The dose should be, for a child three years old, one-sixteenth to one-twelfth of a grain in a teaspoonful of elixir of bismuth

6

and pepsin every three hours, and for an adult one-twelfth to one-eighth of a grain every three hours. The remedy rarely disturbs the stomach, does not produce ptyalism and seldom acts on the bowels. Under its use, commenced early, an ordinary case is convalescent in three days, and it rarely needs to be given longer than five days.

In croup the chloride of gold acts as a specific. It should be given in solution in distilled water. As it is very deliquescent and difficult to weigh, the druggist may dissolve the contents of a fifteen-grain bottle in fifteen drachms of distilled water, and to a child five years old, one to three drops of this may be given in water every one to three hours. In other words, the dose may be one-fiftieth to one-twentieth of a grain. It should not be administered in silver or other metal spoons, but dropped into a glass with a little water. Remarkable effects are reported.

